# Human Cytomegalovirus Antigen Presentation by HLA-DR+ NKG2C+ Adaptive NK Cells Specifically Activates Polyfunctional Effector Memory CD4+ T Lymphocytes

**DOI:** 10.3389/fimmu.2019.00687

**Published:** 2019-04-03

**Authors:** Marcel Costa-García, Michelle Ataya, Manuela Moraru, Carlos Vilches, Miguel López-Botet, Aura Muntasell

**Affiliations:** ^1^Department of Experimental and Health Sciences, University Pompeu Fabra, Barcelona, Spain; ^2^Hospital del Mar Medical Research Institute (IMIM), Barcelona, Spain; ^3^Immunogenetics and HLA Laboratory, Instituto Hospital Universitario Puerta de Hierro, Majadahonda, Spain

**Keywords:** human, natural killer cell, cytomegalovirus, HLA-DR, NKG2C, CD4 T cells

## Abstract

Natural killer (NK) cells play a dual role in the defense against viral pathogens by directly lysing infected cells as well as by regulating anti-viral T cell immunity. Infection by human cytomegalovirus (HCMV) promotes a persistent expansion of NKG2C+ adaptive NK cells which have been shown to display enhanced antibody-dependent responses against infected targets and associated to viral control in transplanted patients. Based on gene expression data showing increased transcription of CIITA and several genes related to the MHC class II pathway in adaptive NK cells, we explored their putative capacity for antigen presentation to CD4+ T cells. Phenotypic analysis confirmed a preferential steady-state expression of HLA-DR by circulating NKG2C+ adaptive NK cells in healthy individuals. Expression of HLA-DR in NKG2C+ adaptive NK cells was variable and unrelated to the expression of activation (i.e., CD69 and CD25) or differentiation (i.e., FcRγ chain, CD57) markers, remaining stable over time at the individual level. Incubation of purified NK cells with HCMV complexed with serum specific antibodies induced an up-regulation of surface HLA-DR concomitant to CD16 loss whereas no changes in CD80/CD86 co-stimulatory ligands were detected. In addition, surface CX3CR1 decreased upon antigen-loading while HLA-DR+ NK cells maintained a CCR7-, CXCR3^low^ homing profile. Remarkably, HCMV-loaded purified NK cells activated autologous CD4+ T cells in an HLA-DR dependent manner. The fraction of T lymphocytes activated by antigen-loaded NK cells was smaller than that stimulated by monocyte-derived dendritic cells, corresponding to CD28-negative effector-memory CD4+ T cells with cytotoxic potential. Antigen presentation by NK cells activated a polyfunctional CD4+ T cell response characterized by degranulation (CD107a) and the secretion of Th1 cytokines (IFNγ and TNFα). Overall, our data discloses the capacity of NKG2C+ adaptive NK cells to process and present HCMV antigens to memory CD4+ cytotoxic T cells, directly regulating their response to the viral infection.

## Introduction

HCMV is a β-herpesvirus that establishes a highly prevalent and generally asymptomatic life-long persistent infection in immunocompetent individuals, alternating subclinical reactivations and latency periods (1). HCMV infection is the leading infectious etiology of congenital sensorineural disabilities and an important co-morbidity in immunocompromised individuals (2–4). HCMV infection control involves the participation of specific T lymphocytes, antibodies, and NK cells, being an environmental factor significantly influencing the configuration of the immune system at individual level (5). Relatively high proportions of HCMV-specific CD8+ and CD4+ T cells are found in peripheral blood of healthy HCMV+ individuals and tend to increase in the elderly, presumably as a result of a continuous virus-host interaction (6–9). HCMV seropositivity is associated to phenotypic and functional changes in specific CD4+ T cells with the variable expansion of an effector-memory population showing a Th1 cytokine profile and the loss of costimulatory molecules (CD28, CD27) concomitant to the acquisition of cytotoxic capacity (10–14). On the other hand, HCMV induces in some individuals a stable adaptive expansion of an NK cell subset, characterized by high surface levels of the activating receptor CD94/NKG2C in the absence of its inhibitory counterpart CD94/NKG2A (15). Differentiation of HCMV-adaptive NK cells is a progressive process which shapes their phenotypic and functional profile, involving the epigenetic regulation of transcription factors and signaling molecules. Adaptive NK cells preferentially express inhibitory killer Ig-like receptor (KIR) specific for self-HLA-C along with reduced NKp30, NKp46, and CD161 surface levels, and include high proportions of LILRB1+, CD57+, and FcεRIγ- cells (15–19). Functionally, NKG2C+ adaptive NK cells are proficient effectors, showing enhanced cytokine secretion (i.e., TNFα and IFNγ), cytotoxic potential (granzyme B) and antibody-dependent anti-viral responses (20–22). Expansions of NKG2C+ adaptive NK cells in kidney transplant recipients have been associated to a lower incidence of post-transplant HCMV viremia, indirectly suggesting that they may be involved in controlling viral reactivation (23).

A relative enrichment for distinct MHC class II-related transcripts, including the master transactivator CIITA, were detected in adaptive NK cells (24, 25). Generally, HLA class II molecules are constitutively expressed by professional APC (e.g., dendritic and B cells), yet can be induced upon activation in a variety of other cell types, including T and NK cells (26). Co-expression of HLA-DR and activation markers (i.e., CD69, CD11c) in circulating NK cells was described in patients with HIV-caused immunodeficiency (27), multiple sclerosis (28), or systemic lupus erythematosus (29). In healthy individuals, HLA-DR expression has been described in CD56^bright^ NK cells (30, 31), albeit relatively high levels of HLA-DR were also observed in CD56^dim^ NK cells from some individuals. Few studies have evaluated HLA class II function on NK cells in the context of superantigen-dependent T cell activation (32), mixed lymphocyte reactions (33), or using synthetic peptides derived from immunodominant antigens (34). Recent studies reported a regulatory role for non-conventional HLA class II expression in steady-state type 2 and 3 innate lymphoid cell subsets (35, 36).

In the present study we have characterized the expression of HLA class II molecules by circulating adaptive NK cells in healthy individuals and their function as non-conventional antigen presenting cells (APC). We showed that NKG2C+ adaptive NK cells can present HCMV-derived antigens through HLA-DR to specific CD4+ T cells, a process that is enhanced by the presence of specific antibodies. Our results reveal a novel mechanism potentially involved in the crosstalk between adaptive NK cells and specific memory CD4+ T cells along persistent HCMV infection.

## Materials and Methods

### Subjects and Ethics Statement

PBMC and serum samples used in this study were obtained from volunteer healthy adults. HCMV seropositive individuals showing ≥20% NKG2C+NKG2A- NK cells were considered to display adaptive NK cell expansions in contrast to seropositive donors with <5% NKG2C+NKG2A- cells in their NK cell repertoire, considered to lack HCMV-adaptive expansions. Written informed consent was obtained from every donor, and the study protocol was approved by the local ethics committee (Clinical Research Ethics Committee, Parc de Salut Mar n°2013/5470/I).

### Antibodies and Immunophenotyping by Flow-Cytometry

FACS analysis was performed using mAbs specific for the following molecules: HLA-DR-fluorescein isothiocyanate (FITC), CD86-FITC, CD45RA-FITC, Perforin-FITC, CD69-Phycoerythrin (PE), CD80-PE, IFNγ-PE, CD4-allophycocyanin (APC), CD3-peridinin-chlorophyll protein (PerCP), CCR7-PE-Cy7, CD16-PE-Cy7, CD8-V500, CD28-PE-CF594 (BD Biosciences, San Diego, CA), CD56-APC, CD25-PE, CX3CR1-PE-Cy7, CXCR3-eFluor 660 (eBioscience, San Diego, CA), NKG2C-PE (clone 134591), NKG2C-Alexa Fluor 700 (clone 134591) and unlabeled-NKG2C (clone MAB1381; R&D Systems, Minneapolis, MN), anti-FcεRI Ab, γ subunit-FITC (Merck, Millipore), CD4-FITC, CD4-PE-Cy7, Granzyme B-Pacific Blue (PB; Biolegend, San Diego, CA), and NKG2D-APC (Miltenyi Biotec, Bergisch Gladbach, Germany). Anti–TNF-α (infliximab; REMICADE) was directly labeled with CF-Blue by Immunostep (Salamanca, Spain). Anti-NKp46 (clone Bab281) and anti-NKp30 (AZ20) mAbs were kindly provided by Dr. A. Moretta (University of Genova, Genova, Italy); anti-CD57 (clone HNK-1), anti-LILRB1 (clone HP-F1), and anti-CD161 (clone HP-3G10) were produced in our laboratory and employed as hybridoma culture supernatants. Cells were pre-treated with human aggregated IgG (10 μg/ml) to block Fc receptors and subsequently labeled with specific Abs. For indirect immunostaining, samples were incubated with primary Abs followed by PE-Cy7-conjugated or APC-Cy7-conjugated F(ab′)2 polyclonal goat antimouse IgG (Biolegend). Samples were acquired in LSRII or LSRFortessa flow cytometers (BD Biosciences), and data analyzed with FlowJo software (Tree Star). For blocking experiments, the anti-HLA-DR D1.12, kindly provided by Dr. R. Accolla (Università of Insubria, Varese) or an isotype control were used at saturating concentration. Mean Fluorescence Index for HLA-DR was calculated as previously described (37) using the following formula: mean fluorescence positive–mean fluorescence negative control/(2 × Standard Deviation mean fluorescence negative control).

### HCMV Stock Preparation

The MRC5 fetal human lung fibroblast cell line was obtained from the American Type Culture Collection (Manassas, VA) and grown in DMEM supplemented with 10% fetal bovine serum (FBS), penicillin, and streptomycin. Purified stocks of HCMV AD169 strain were prepared by infecting MRC5 cells at 0.25 multiplicity of infection (MOI) and harvesting supernatants when maximum cytopathic effect was reached. Cells and debris were removed from virus containing supernatant by centrifugation 10 min at 5000 x g and stored at −80°C. Viral stocks were titrated on MRC5 cells analyzed by detection of IE-1/IE-2 viral antigens with specific mAb (clone mab810; Millipore) by immunofluorescence as previously described (38).

### Primary NK Cell and CD4+ T Cell Purification

Peripheral blood mononuclear cells (PBMC) were obtained from heparinized blood samples by separation on Ficoll-Hypaque gradient (Lymphoprep; Axis-Shield PoC AS, Oslo, Norway). Serum samples were collected, heat-inactivated and aliquoted before storage at −20°C. Standard clinical diagnostic tests were used to determine HCMV specific IgG titer (Roche Diagnostics, Basel, Switzerland). PBMC were kept overnight with complete RPMI 1640 medium supplemented with 200 U/ml of recombinant human interleukin-2 (rhIL-2; Proleukin, Chiron, Emeryville, CA prior to proceeding with NK cell, or CD4+ T cell purification. NK cells were purified by negative selection using NK Cell Isolation kit (Miltenyi) according to the manufacturer instructions. Of note, some of the commercially available kits for NK cell isolation through negative selection include anti-HLA-DR antibodies resulting in the depletion of HLA-DR+ NK cells from the isolated pool, as also observed by Kovalenko et al. (39).

Autologous CD4+ T cells purified by negative selection using the CD4+ T cell Isolation Kit (Miltenyi) or PBMC (5:2 E:T ratio) were used as effector cells in functional assays.

Monocyte-derived dendritic cells (moDCs) were generated as previously described (40); briefly, monocytes were positively selected from fresh PBMCs using anti-CD14 microbeads (StemCell Technologies, Grenoble, France), and cultured for 6 days in RPMI 1650 medium supplemented with 10% FBS, interleukin-4 (IL-4; 25 ng/ml, R&D Systems), and granulocyte-macrophage colony-stimulating factor (GM-CSF; 50 ng/ml, PeproTech).

### CD4+ T Cell and NK Cell Expansions

HCMV-specific CD4+ T cells were expanded by incubating PBMC with HCMV virion preparations (2 × 10^5^ PFU/3 × 10^6^ cells) in 24-well plates. Cell cultures were maintained at 37°C in a 5% CO_2_ humid atmosphere for 10–12 days. At day 3, cell cultures were supplemented with 25 U/mL of rhIL2 and half of the supernatant was replaced with fresh rhIL2-containing medium every 3 days; proliferating cell cultures were eventually split when required.

NK cells were expanded by incubating PBMC with irradiated HLA-E+ 721.221-AEH lymphoblastoid cell line (41) in 24-well plates (3:1 ratio) in complete RPMI 1640 medium. Cell cultures were maintained at 37°C in a 5% CO_2_ humid atmosphere for 10–12 days; every 3 days half of the supernatant was replaced with fresh medium; when high cell density was attained, cell cultures were split. Expanded NK cells were further purified using the corresponding enrichment kit.

### Antigen-Presentation Assays

NK cells or moDCs were cultured overnight with titrated HCMV preparations at MOI 2.5 in the presence or absence of 10% sera from HCMV+ donors. Subsequently, antigen-loaded or control APCs were incubated with autologous CD4+ T cells or PBMC (5:2 E:T ratio) for 18 h at 37°C in the presence of Brefeldin A (10 μg/ml; Sigma-Aldrich). Next, cells were stained with antibodies recognizing surface markers, fixed, permeabilized (fixation/permeabilization kit; eBioscience), stained with anti-TNFα and anti-IFNγ, and analyzed by flow cytometry. In some experiments, CD4+ T cell degranulation was monitored by measuring CD107a mobilization with the additional presence of monensin (5 μg/ml; Sigma-Aldrich) and CD107a-FITC (BD Biosciences Pharmingen, San Diego, CA). Boolean gating function was used to identify all possible combinations of markers stained for on CD3+ CD4+ T cell populations (Flowjo software). In antigen presentation assays, 122,400 ± 31,907 total CD3+ CD4+ T cells and 1,029 ± 293 of activated CD3+ CD4+ T cells were acquired (mean ± SEM). In some experiments, chloroquine (50 μM) was added along NK cell incubation with HCMV preparations. NK cells were incubated with pp65 and IE1 overlapping peptide mixtures as control in some antigen presentation experiments (PepTivator CMV pp65 human, PepTivator CMV IE-1 human, Miltenyi).

### Statistical Analysis

Statistical analysis was performed by the Mann Whitney U test using GraphPad Prism 5 software. Results were considered significant at the two-sided P level of 0.05.

## Results

### HLA-DR Is Detected in Circulating NKG2C+ Adaptive NK Cells Uncoupled From Activation and Differentiation Markers

**Figure 1:**
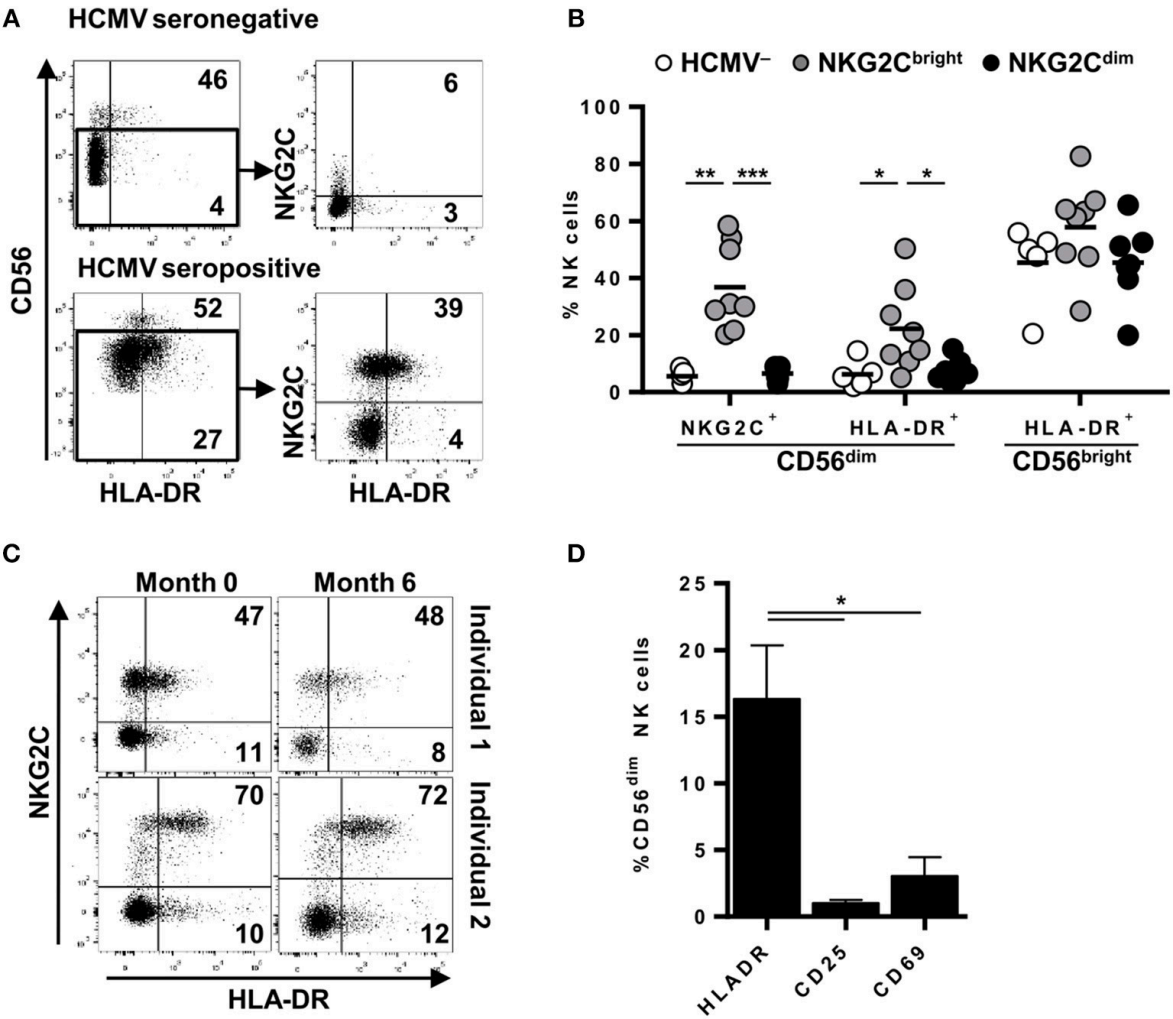
Surface expression of HLA-DR is stably detected in variable proportions of circulating NKG2C+ adaptive NK cells in the absence of activation markers. NKG2C and HLA-DR expression was analyzed by flow cytometry in circulating NK cells from seronegative (*n* = 5; HCMV–) and seropositive (HCMV+) individuals with (*n* = 8; NKG2C^bright^) or without (*n* = 7; NKG2C^dim^) NKG2C+ adaptive NK cells. **(A)** Representative dot plots of NKG2C and HLA-DR expression in CD56^dim^ NK cells from HCMV- and HCMV+ individuals. Inset numbers indicate proportions of HLA-DR+ in CD56^bright^ and CD56^dim^ gates. **(B)** Percentage of NKG2C+ and HLA-DR+ cells in CD56^dim^ and CD56^bright^ NK cell subsets in individuals categorized according to their HCMV serology and the presence (NKG2C^bright^) or absence (NKG2C^dim^) of NKG2C+ adaptive NK cells. **(C)** Dot plots showing NKG2C and HLA-DR phenotype along time in two out of five HCMV+ individuals analyzed. Inset numbers indicate frequencies of HLA-DR+ cells in NKG2C+ and NKG2C- NK cells. **(D)** HLA-DR, CD25, and CD69 expression on circulating CD56^dim^ NK cells from HCMV+ individuals with NKG2C+ adaptive NK cells (mean ± SEM, *n* = 6) (*p < 0.05, **p < 0.01, ***p < 0.001).

The analysis of published transcriptional programs of adaptive NK cells, defined as CD56^dim^ NKG2C+ (CD57+/FcεRIγ-) (24, 25), identified transcripts for CIITA, HLA-DQ, HLA-DP, HLA-DMA, and HLA-DRA to be enriched in this NK cell subset (Supplementary Figure 1). In order to ascertain the predicted expression of MHC class II molecules on adaptive NK cells, we analyzed by flow cytometry HLA-DR in circulating NK cells from healthy individuals, stratified by the presence or absence of NKG2C+ adaptive NK cell expansions, according to the criteria described in Materials and Methods. As shown in Figure 1, HLA-DR was expressed in approximately ~50% of circulating CD56^bright^ NK cells in all analyzed donors. In contrast, the proportions of HLA-DR+ CD56^dim^ NK cells varied in different individuals and were generally higher in HCMV+ donors coinciding with the expansion of NKG2C+ adaptive NK cells, as compared to individuals lacking this phenotype regardless of their HCMV serostatus (Figures 1A–B and Supplementary Figure 2). Of note, proportions of HLA-DR+ NKG2C+ NK cells remained stable over time (Figure 1C) and were unrelated to the expression of activation markers (i.e., CD69 and CD25) (Figure 1D).

**Figure 2:**
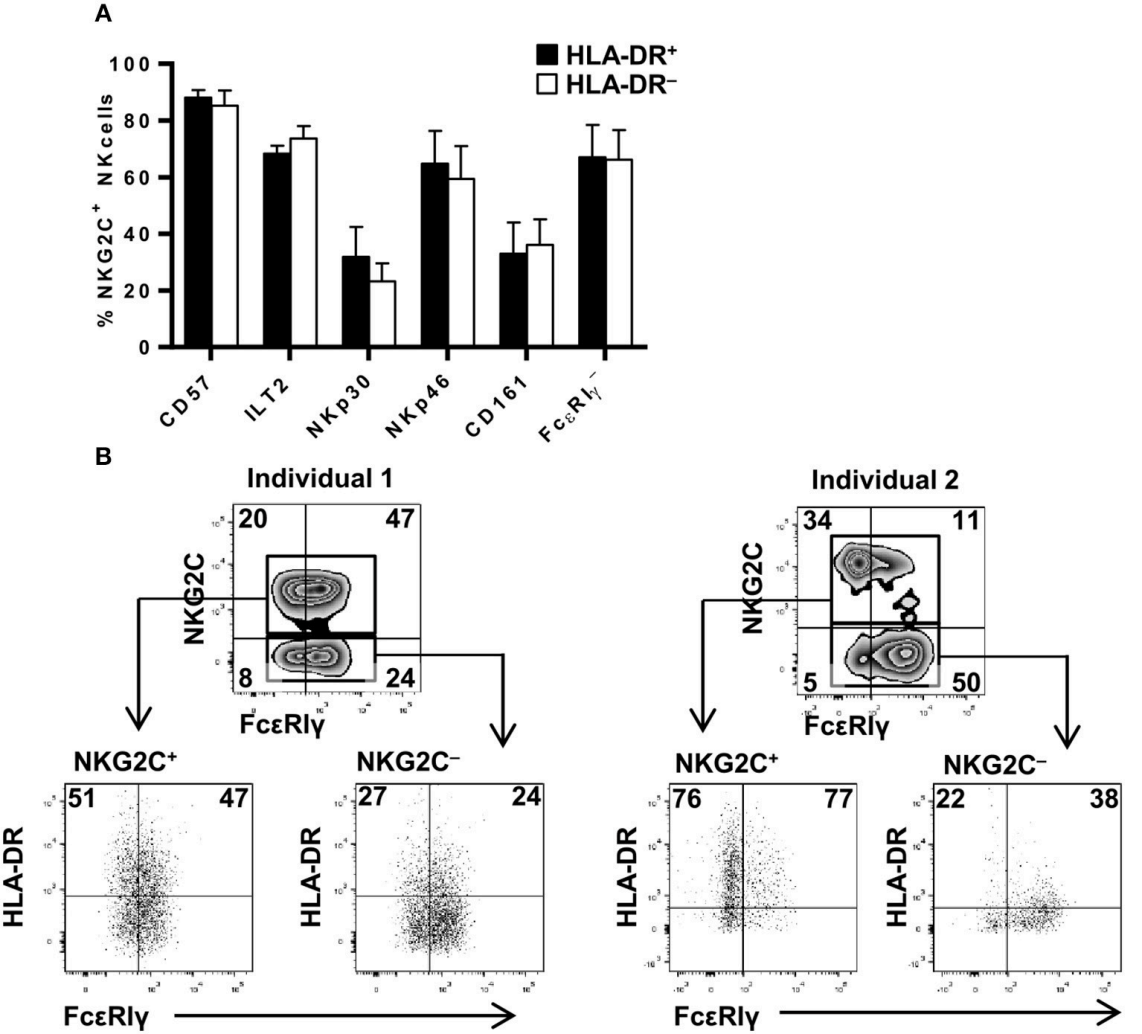
HLA-DR expression in NKG2C+ adaptive NK cells is uncoupled from phenotypic features associated to their differentiation profile. The expression of FcεRIγ, NKp30 and NKp46 NCRs, CD161, CD57, and ILT2 (LILRB1) was analyzed in NKG2C+ HLA-DR+ and NKG2C+ HLA-DR– circulating NK cells from seropositive individuals with NKG2C+ adaptive NK cell expansions. **(A)** Percentage of CD57, ILT2, NKp30, NKp46, CD161 positive, and FcεRIγ negative cells in CD56^dim^ NKG2C+ NK cells according to HLA-DR co-expression (mean ± SEM, *n* = 5). **(B)** Expression of HLA-DR and FcεRIγ in NKG2C+ and NKG2C– adaptive NK cells from two representative donors out of five studied. Inset numbers in lower panels indicate the proportions of HLA-DR in FcεRIγ + and FcεRIγ-NK cells.

HCMV-adaptive NKG2C+ NK cells have been proposed to undergo a sequential differentiation associated to the down-regulation of FcεRIγ, NKp30, NKp46, and CD161 expression and the acquisition of CD57 and LILRB1 (16, 20, 42). Since proportions of HLA-DR+ NKG2C+ adaptive NK cells varied between different individuals, we analyzed whether expression of HLA-DR coincided with the acquisition of a specific differentiation molecular signature. Expression of KIR, CD57, LILRB1, NKp30, NKp46, CD161, and FcεRIγ and HLA-DR was analyzed in NK cells from five HCMV+ individuals displaying NKG2C+ adaptive NK cell expansions. The distribution of all assessed markers was comparable in HLA-DR+ and HLA-DR– NKG2C+ adaptive NK cells (Figure 2A). NKG2C-negative adaptive NK cell expansions have also been previously characterized for their oligoclonal KIR expression profile (17) and/or the loss of signaling adaptors such as FcεRIγ chain (20, 24, 43). Detailed analysis of HLA-DR expression in two individuals concomitantly displaying NKG2C+ and NKG2C– FcεRIγ- NK cell subpopulations confirmed the preferential expression of HLA-DR in adaptive NKG2C+ NK cells independently of FcεRIγ levels in these cases (Figure 2B). Altogether, these results indicate that HLA-DR expression in NKG2C+ adaptive NK cells occurs dissociated from other differentiation/adaptive features.

### Sensing of HCMV-antibody Immune Complexes Upregulates HLA-DR in NKG2C+ Adaptive NK Cells in the Absence of CD80/CD86 Expression

We have previously shown that NK cells can directly sense the presence of HCMV virions and HCMV-antibody immune complexes (IC) (21, 44). We next addressed whether co-culture of primary NK cells with these stimuli could lead to HCMV antigen presentation by HLA class II molecules. To address this question purified NK cells were cultured overnight with HCMV (AD169 strain at MOI 2.5), including or not serum from seropositive donors. For comparison, autologous moDC were cultured in parallel in the same conditions. Incubation with HCMV did not result in NK cell or moDC infection, assessed by IE-1/IE-2 expression (not shown).

**Figure 3:**
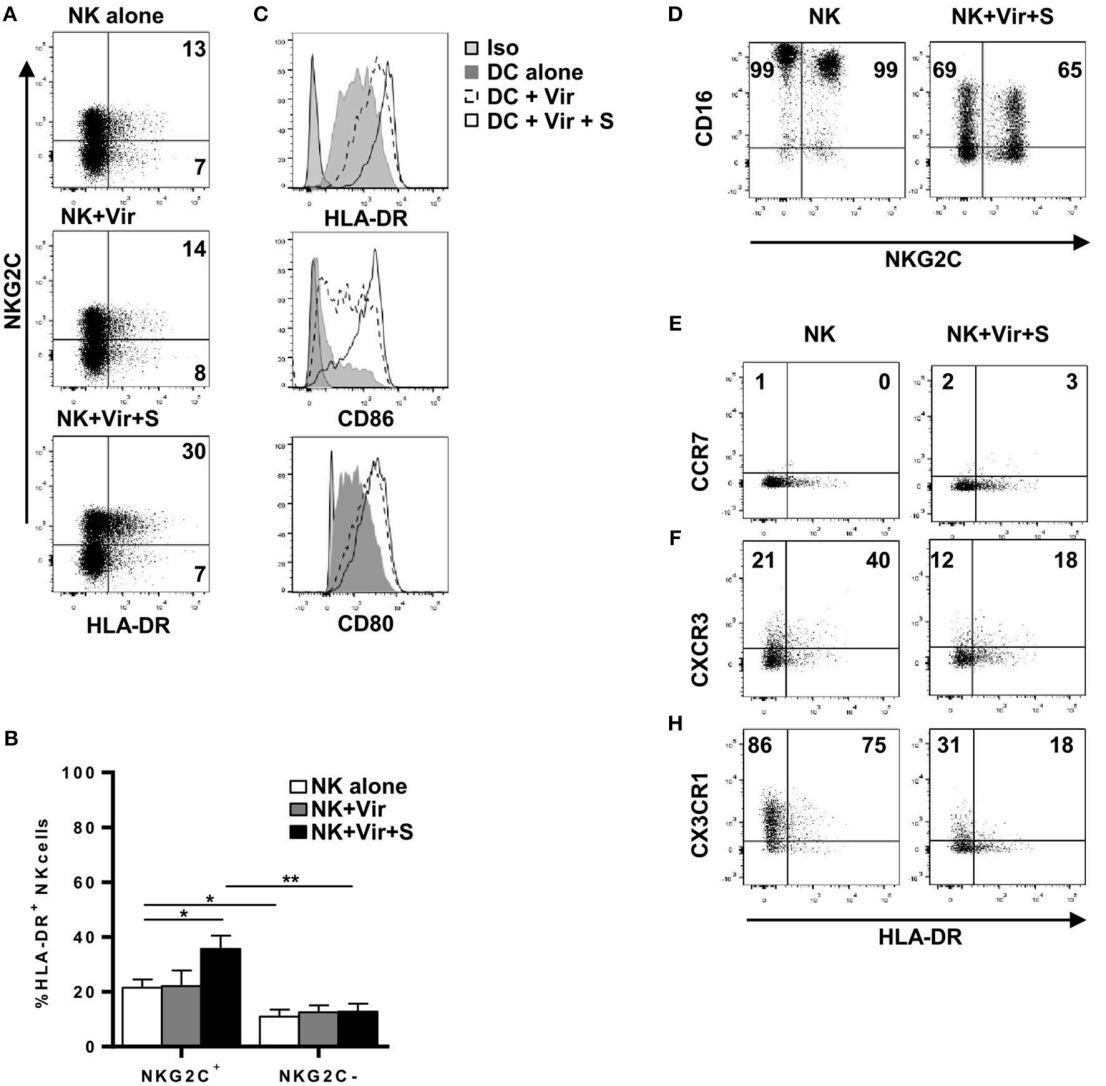
NKG2C^bright^ NK cells up-regulate HLA-DR upon HCMV antigen uptake in the presence of specific antibodies. NK cells and moDC were cultured 20 h with HCMV viral particles in the presence or absence of serum from HCMV+ donors. Expression of HLA-DR, CD86, CD80, CD16, CCR7, CXCR3, and CX3CR1 was analyzed by flow cytometry. **(A)** Dot plots of HLA-DR and NKG2C expression in NK cells in the indicated conditions. Inset numbers indicate the frequency of HLA-DR+ cells in NKG2C+ and NKG2C- NK cells. Data from a representative donor out of four studied. **(B)** Bar graph showing the average expression of HLA-DR in NKG2C+ and NKG2C– NK cells in the different conditions (mean ± SEM, *n* = 4) (*p < 0.05, **p < 0.01). **(C)** Histograms displaying HLA-DR, CD86, and CD80 expression in moDC in the indicated conditions. **(D)** Dot plot showing CD16 and NKG2C expression in NK cells incubated or not with HCMV virions and specific serum. Inset numbers indicate percentages of CD16+ cells in NKG2C+ and NKG2C- NK cells. **(E–H)** Dot plot showing CCR7, CXCR3, and CX3CR1 in HLA-DR+ and HLA-DR- NK cells incubated or not with HCMV virions and specific serum. Data from one donor out of three analyzed are shown. Inset numbers indicate percentages of cells positive for each chemokine receptor in NKG2C+ and NKG2C- NK cells.

Up-regulation of surface HLA-DR, CD80, and CD86 in moDC was detectable following overnight co-culture with HCMV preparations, yet no significant changes were noticed in NK cells. In contrast, stimulation with HCMV in the presence of HCMV+ serum promoted an up-regulation of surface HLA-DR in both NK and moDC, enhancing CD80/86 expression in the latter (Figures 3A,B and not shown). Among CD56^dim^ NK cells, enhancement of surface HLA-DR expression was more evident in the NKG2C+ adaptive subset in concordance with their higher baseline expression (Figure 3C). Of note, a reduction of surface CD16 (Figure 3D) and the production of TNFα (not shown) (21) was detected upon overnight culture indicating NK cell sensing of HCMV-antibody immune complexes. Overnight incubation with HCMV immune complexes did not alter CCR7 expression, though surface CXCR3 and CX3CR1 was reduced in CD56^dim^ NK cells after antigen loading (Figures 3E–H).

### HCMV Antigen-Loaded NK Cells Activate HCMV-Specific CD4+ T Cells in an HLA-DR-Dependent Manner

**Figure 4:**
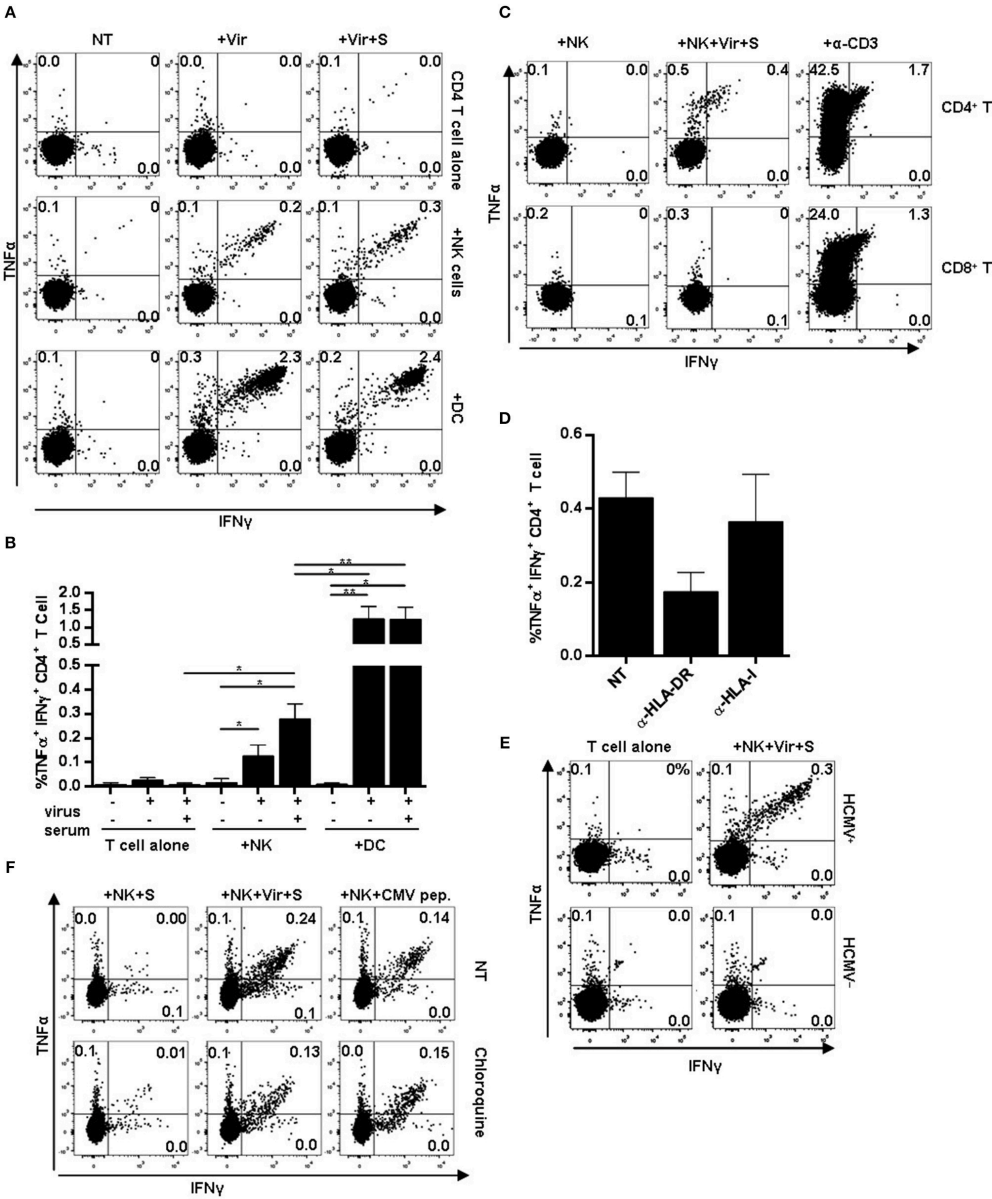
CD4+ T cell activation in response to HCMV antigen presentation by NK or moDC. NK cells or moDCs previously loaded with HCMV-antibody immune complexes were cultured overnight with autologous CD4+ T cells. TNFα and IFNγ production was analyzed by flow cytometry. **(A)** TNFα and IFNγ production by CD4+ T cells in the indicated conditions. Data from a representative donor out of five tested. **(B)** Mean frequency of IFNγ+ and TNFα+ CD4+ T cells upon activation with different APCs (mean ± SEM, *n* = 5) (*p < 0.05, **p < 0.01). **(C–E)** Autologous PBMC were used as effectors in co-culture experiments with NK cells pre-incubated with HCMV-antibody immune complexes. CD4+ and CD8+ T cell activation was analyzed by flow cytometry. An agonist anti-CD3 antibody was used as a positive control. **(C)** Dot plots display intracellular TNFα and IFNγ in CD4+ and CD8+ T cells in the indicated conditions. Data from a representative donor out of four analyzed. **(D**) Frequency of TNFα+ and IFNγ+ CD4+ T cells in response to HCMV-loaded NK cells in the presence of blocking antibodies specific for HLA-DR and HLA class I molecules (mean ± SEM, *n* = 3). **(E)** TNFα and IFNγ intracellular staining of CD4+ T cells in co-cultures including antigen-presenting NK cells and autologous PBMC from HCMV seropositive and seronegative individuals. **(F)** Frequency of TNFα+ and IFNγ+ CD4+ T cells in response to HCMV-loaded NK cells. NK cells were loaded in the presence or absence of chloroquine (50 μM). Dot plots of one out of two donors tested.

Purified primary NK cells from individuals with NKG2C+ adaptive NK cell expansions and moDCs were pre-incubated overnight with HCMV in the presence or absence of HCMV+ sera and subsequently used as APCs in co-cultures with autologous primary CD4+ T lymphocytes. CD4+ T cell activation was monitored through the production of intracellular TNFα and IFNγ at 20 h by flow cytometry (Figure 4). Co-culture with autologous CD4+ T lymphocytes did not promote cytokine production by NK cells (not shown). However, NK cells pre-incubated with HCMV viral particles triggered the activation of a small fraction of CD4+ T lymphocytes, as detected by the simultaneous production of TNFα and IFNγ; higher proportions of CD4+ T cells were activated by NK cells antigen-loaded in the presence of HCMV+ donor serum (Figures 4A,B). The average proportion of CD4+ T cells activated by virus-loaded moDC was 10-fold higher than that induced by antigen-presenting NK cells, and was not enhanced by HCMV+ sera (Figures 4A,B). Of note, in functional assays including autologous PBMC as effectors, only CD4+ but not CD8+ T cell activation could be detected upon co-culture with HCMV-loaded NK cells (Figure 4C). In fact, NK cell-induced CD4+ T cell activation was partially blocked by an α-HLA-DR antibody (Figure 4D) and could not be detected in experiments with cells from HCMV-seronegative donors, emphasizing the importance of HLA-DR in antigen presentation and supporting the requirement of an expanded pool of antigen-experienced CD4+ T cells (Figure 4E). The addition of chloroquine, an inhibitor of endosomal and lysosomal acidification, along NK cell-loading with HCMV, partially prevented CD4+ T cell activation. In contrast, presentation of peptide mixtures spanning pp65 and IE-1 immunodominant HCMV antigens to CD4+ T cells was unaffected by the drug (Figure 4F). Of note, analysis of the HLA class II genotype evidenced that several of the individuals showing expansions of HLA-DR+ NKG2C+ NK cells expressed HLA class II alleles previously shown to present HCMV immunodominant peptides (i.e., HLA-DR7) (Supplementary Table I) (45).

Overall, these data indicate that circulating NK cells can process HCMV particles and present peptides by MHC class II to antigen-primed CD4+ T cells in a process that may be enhanced by stimulation with HCMV-antibody complexes.

### HCMV-Specific CD4+ T Cells Activated by Antigen-Loaded NK Cells Display an Effector Memory Phenotype, Lack CD28 Expression and Have Cytotoxic Potential

**Figure 5:**
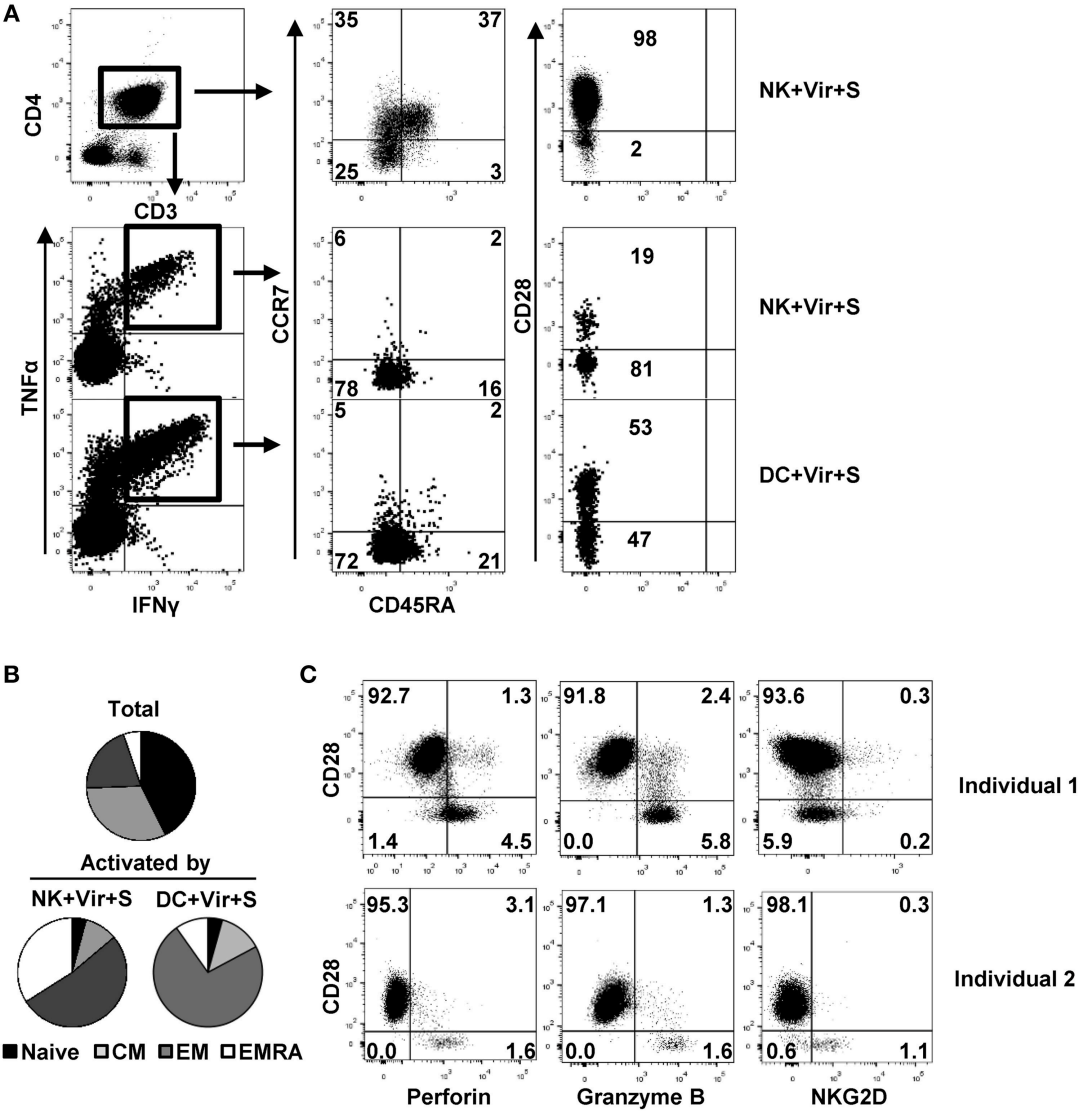
Differentiation and functional profile of HCMV-specific CD4+ T cells activated by antigen-presenting NK cells. NK cells or moDC previously loaded with HCMV in the presence of specific antibodies were cultured with autologous CD4+ T cells and the production of TNFα and IFNγ in combination with CD45RA, CCR7, and CD28 differentiation markers was analyzed by multiparametric flow cytometry. **(A)** Dot plots showing CD45RA, CCR7, and CD28 expression in total and activated (IFNγ+ TNFα+) CD4+ T cells from a representative donor out of four in the indicated conditions. **(B)** Pie chart showing the distribution of CD4+ T cell subpopulations based on CCR7 and CD45RA at baseline and of those T cells activated by antigen-presenting NK cells and DC (*n* = 4). **(C)** Perforin, granzyme B, and NKG2D expression in CD28+ and CD28– CD4+ T cells from two representative HCMV+ individuals out of three analyzed.

We analyzed the differentiation profile of CD4+ T cells activated in response to antigen presenting NK cells by monitoring the expression of CCR7, CD45RA, and of CD28 as previously defined (14, 46). CCR7 and CD45RA expression define four T cell populations: naïve (CD45RA+ CCR7+), central memory (CD45RA– CCR7+), effector memory (CD45RA– CCR7–) and terminally differentiated effector memory (TEMRA) (CD45RA+, CCR7–) T cells, whereas CD28 negative circulating CD4+ T cells have been identified in individuals with chronic/persistent viral infections (e.g., HCMV) (11, 14). The majority of CD4+ T cells activated in response to HCMV-loaded NK cells displayed an effector memory (EM) or TEMRA phenotype, lacking CD28 (Figures 5A,B). In contrast, both CD28+ and CD28– effector memory CD4+ T cells were activated upon co-culture with HCMV-loaded moDC (Figures 5A,B). Thus, activation of CD28– memory CD4+ T cells by antigen presenting NK cells likely reflects their capacity for responding to lower HLA class II-peptide levels in the absence of CD28 co-stimulatory signaling. In agreement with previous reports (11, 14), CD28– CD4+ T cells in HCMV seropositive donors were perforin+ and granzyme B+, with variable co-expression of the activating NK cell receptor NKG2D (Figure 5C) (47).

**Figure 6:**
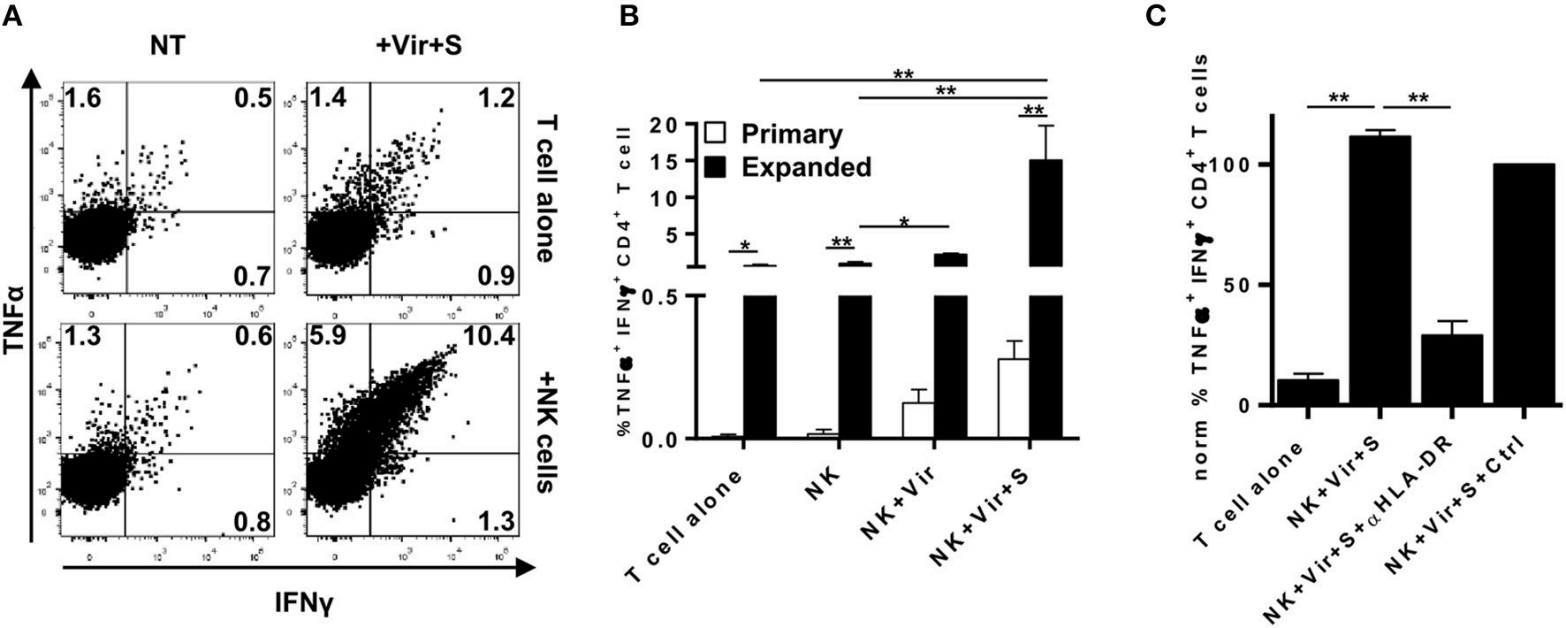
Antigen presentation by expanded HLA-DR+ NKG2C+ NK cells to HCMV-specific autologous CD4+ T cell lines. Expanded NKG2C+ NK cells pre-loaded with HCMV in the presence of immune serum were cultured overnight with HCMV-expanded CD4+ T cells. Intracellular TNFα and IFNγ was analyzed by flow cytometry. **(A)** TNFα and IFNγ in CD4+ T cells cultured in the indicated conditions. Data from a representative donor. **(B)** Comparison of the frequency of TNFα+ IFNγ+ CD4+ T cells in co-culture experiments using primary or expanded antigen presenting and effector cells (mean ± SEM, *n* = 5) (*p < 0.05, **p < 0.01). **(C)** Frequency of TNFα+ IFNγ+ HCMV-expanded CD4+ T cells upon co-culture with antigen-loaded NK cells in the presence of an α-HLA-DR (clone D1.12) or an isotype control (mean ± SEM, *n* = 5).

An assay employing autologous expanded HCMV-specific CD4+ T cells and HLA-DR+ NKG2C+ NK cells was set up to enhance the sensitivity of the experimental system. HCMV-specific CD4+ T cells were enriched by culturing PBMC from HCMV seropositive individuals with HCMV viral particles in the presence of IL-2 as previously described (47). Under these conditions, expanded CD4+ T cells presented an effector-memory phenotype, high levels of perforin and granzyme B and were mostly CD28 positive yet with variable expression of NKG2D (Supplementary Figure 3). NKG2C+ HLA-DR+ NK cells were expanded in parallel by co-culturing PBMC with the .221-AEH cell line. After 9 days, the majority of expanded NK cells were CD16+, NKG2C+, HLA-DR+, and expressed CD86 (Supplementary Figure 4). NK cell lines were pre-loaded with HCMV particles in the presence or absence of HCMV+ serum and co-cultured with expanded CD4+ T cells. As compared to results with primary lymphocytes, average proportions of CD4+ T cells activated by antigen-loaded NK cells in the absence or presence of HCMV+ serum was 12 to 52-fold higher respectively, reaching up to 10% of total CD4+ T lymphocytes (Figures 6A,B). NK cell-dependent CD4+ T cell activation could be blocked by an anti-HLA-DR antibody (Figure 6C) and, in accordance with the primary setting, no cytokine production was detected in NK cells, ruling out their response against autologous CD4+ T cells (not shown).

### Antigen-Presentation by NK Cells Triggers a Polyfunctional CD4+ T Cell Response

**Figure 7:**
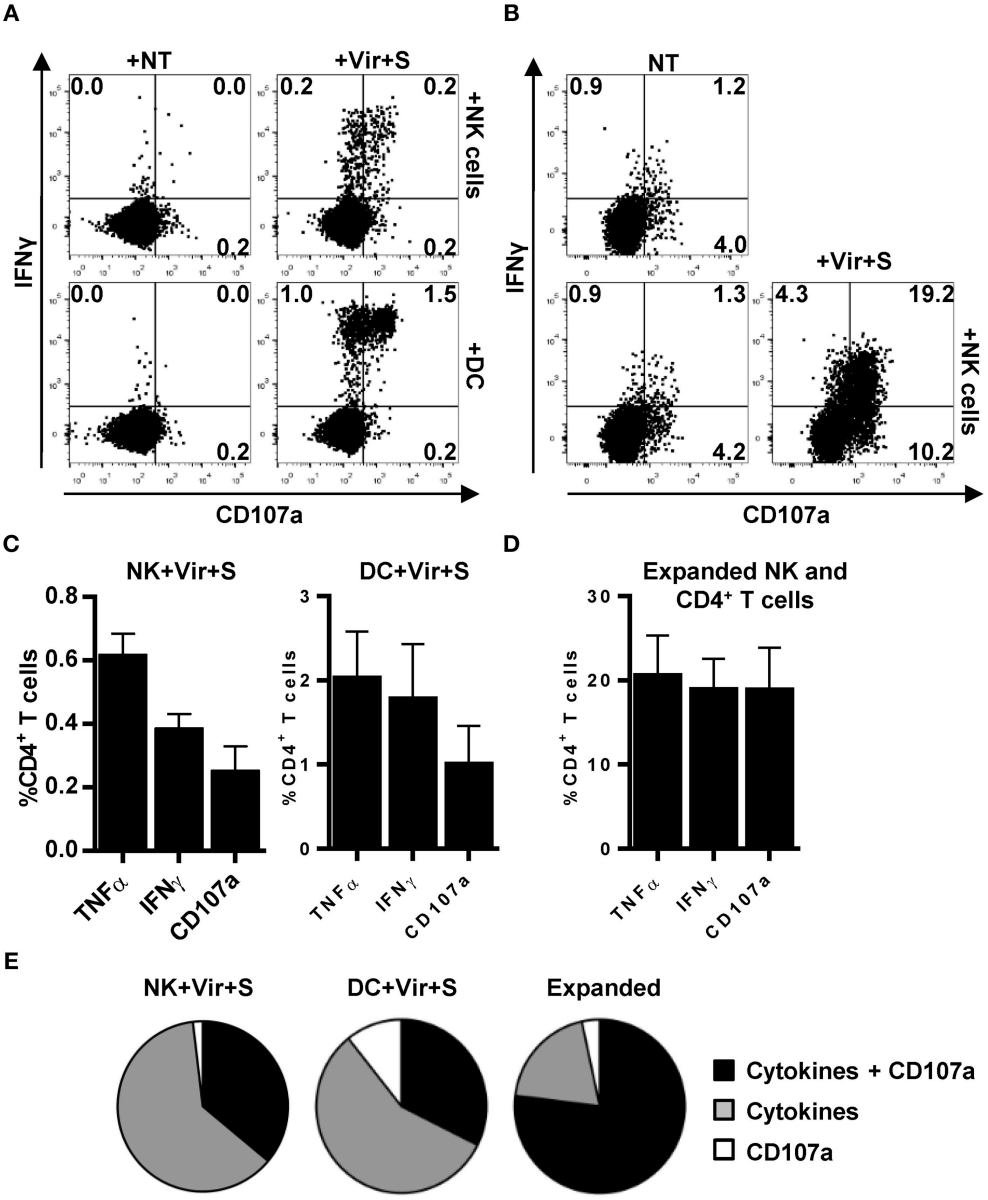
Primary and expanded HCMV-specific CD4+ T cells degranulate in response to antigen loaded NK and moDC. TNFα and IFNγ production concomitant to CD107a mobilization was monitored by flow cytometry in co-culture experiments combining HCMV-loaded primary NK cells or moDC with primary autologous CD4+ T cells **(A,C,E)** or, alternatively, antigen loaded expanded NK cells and HCMV-expanded CD4+ T cells **(B,D,E)**. **(A,B)** Dot plots displaying the proportions of IFNγ+ CD107a+ CD4+ T cells in the primary **(A)** and expanded **(B)** experimental systems. Data from a representative experiment. **(C,D)** Proportions of TNFα+, IFNγ+, and CD107+ CD4+ T cells in the different experimental systems assayed (mean+SEM; *n* = 4). **(E)** Pie charts showing the distribution of CD4+ T cells that produce cytokines and degranulate against HCMV-loaded APC in the indicated experimental systems (mean; *n* = 4).

We next assessed whether CD4+ T cell activation by HCMV-loaded NK cells was qualitatively comparable to that induced by professional APCs. For that purpose, CD4+ T cell degranulation as well as IFNγ and TNFα production were simultaneously analyzed by flow cytometry in co-culture experiments with autologous NK cells or moDC, previously incubated with HCMV-antibody immune complexes. As shown in Figure 7, ~65% of primary CD4+ T cells activated by antigen presenting NK cells secreted TNFα and IFNγ whereas concomitant degranulation was detected in ~35% of them. Antigen presentation by moDC triggered a more heterogeneous CD4+ T cell response including ~15% of cells degranulating in the absence of cytokine production, ~60% of cells producing pro-inflammatory cytokines and ~40% of cells showing a polyfunctional response with concomitant degranulation and cytokine production. In co-cultures using expanded NK and CD4+ T cells up to ~75% of the responding CD4+ T cells degranulated and produced TNFα and IFNγ (Figures 7B–E).

## Discussion

HCMV promotes in some individuals an adaptive reconfiguration of the NK cell compartment characterized by the persistent expansion of a subset of NKG2C+ NK cells (15). These adaptive NK cells display a particular phenotypic and functional profile, efficiently mediating antibody-dependent NK cell responses against virus-infected cells (20–22). Pre-transplant expansions of NKG2C+ adaptive NK cells have been associated to a reduced incidence of HCMV viremia in kidney transplant recipients (23). In the current study, we have analyzed a novel functional feature of NKG2C+ adaptive NK cells related to their expression of HLA class II molecules. Our data demonstrates the preferential and persistent expression of HLA-DR in circulating NKG2C+ adaptive NK cells among the CD56^dim^ subset, as well as their capacity for processing and presenting HCMV antigens to effector memory CD4+ T cells, triggering a polyfunctional Th1/cytotoxic response. HCMV immune complexes enhanced antigen presentation. Whether this process may regulate *in vivo* the development of HCMV-specific memory CD4+ T cell responses, contributing to the control of viral reactivation, deserves attention.

HLA class II expression on a variable fraction of peripheral blood NK cells, mainly coinciding with CD56^bright^ NK cells, had been previously described in healthy individuals (30, 31). The analysis of transcriptional programs in adaptive NKG2C+ NK cells identified MHC class II antigen presentation as an enriched functional pathway in this NK cell subset (24, 25) (Supplementary Figure 1). Our phenotypic studies including selected healthy blood donors with known NK cell receptor repertoires confirmed that HLA-DR+ CD56^dim^ NK cells were more frequently detected in HCMV+ individuals coinciding, though not exclusively, with a variable fraction of NKG2C+ adaptive NK cells. Whether HLA-DR expression on NKG2C+ adaptive NK cells reflected a reversible activation state or was associated with their differentiation was addressed. The fact that surface HLA-DR was uncoupled from the expression of activation markers and co-stimulatory molecules, remaining stable along the follow-up, rather supported its association with a differentiation status of adaptive NK cells. However, HLA-DR expression appeared unrelated to the levels of FcεRIγ and other adaptive NK cell differentiation markers (i.e., CD57 and LILRB1). It is conceivable that epigenetic remodeling associated with adaptive NK cell differentiation (24, 48) might facilitate transcription of CIITA (49) and other HLA class II related genes in a fraction of adaptive NK cells contributing to their functional specialization.

Previous studies addressing HLA class II function on NK cells have tested soluble peptides (33, 34) and Staphylococcal Enterotoxin B crosslinking (32) for triggering HLA-class II-dependent CD4+ T cell activation, hence bypassing the requirement for whole antigen uptake, processing and presentation by the NK cell. Expanded NK cell clones were shown to process and present HLA class II-dependent peptides derived from soluble proteins though failed to present whole *Mycobacterium leprae* (50). The herein presented results showed that NK cells can, indeed, perform these processes upon direct or antibody-aided interaction with viral preparations. It is plausible that our viral preparations contained non-infectious particles or viral antigens facilitating the uptake by a non-professional antigen presenting cell such as NK cells, nonetheless, their decreased antigen presenting function in experiments including chloroquine, indirectly supported their capability for processing and presenting exogenously-added HCMV-derived antigens through HLA class II. Direct viral antigen uptake could be mediated by TLR2 binding with gB and gH HCMV envelope proteins (51), an interaction previously involved in type I IFN production leading to NK cell priming (44). Our data indicate that incubation with HCMV–antibody immune complexes promoted a partial NK cell activation evidenced by low degranulation and the production of TNFα in the absence of IFNγ (21) as well as a partial down-regulation of CD16, concomitant to increased surface HLA-DR levels (Figure 3). The formation of viral antigen-antibody immune complexes could facilitate their uptake prior to CD16 shedding resulting from NK cell activation (52, 53). On the other hand, the increase in HLA-DR surface levels on NK cells in these conditions might also enhance antigen presentation to CD4+ T cells. The capacity of FcγR for enhancing antigen uptake has been extensively described in APCs such as DCs and macrophages (54, 55). In professional APCs antigen uptake through immune complexes enhances antigen presentation and cross-presentation through HLA class II and I respectively, allowing the simultaneous activation of specific CD4+ and CD8+ T cells (56). Our data showed that presence of HCMV-antibody immune complexes enhanced HLA class II-dependent antigen presentation by NK cells though did not appear to detectably enable simultaneous HLA class I-mediated cross-presentation of viral-derived antigens activating CD8+ T cells. Observations in functional assays supported that the absence of co-stimulatory molecules together with their relatively low levels of surface HLA-DR in steady state resulted in an antigen presenting function of adaptive NK cells, yet less fitted in comparison to professional APCs.

The fraction of HCMV-specific CD4+ T cells activated by NK cells was confined into the atypical CD28- effector-memory pool with cytotoxic potential, previously described in association with HCMV infection (11, 12, 57). HCMV-loaded moDCs activated both CD28- and CD28+ effector memory CD4+ T cells likely as a result from presenting a broader virus-derived peptide repertoire in the context of higher HLA class II surface expression and CD80/CD86 co-stimulatory ligands. It is likely that the expression of specific HLA class II molecules capable of presenting immunodominant HCMV antigens such as HLA-DR7 or HLA-DP10 (11, 45, 58) may also influence on the antigen presenting capacity of NK cells, as previously described for other non-professional APCs such as fibroblasts (59). Actually, gB-specific CD4+ T cell responses were found in 95% of healthy donors ranging from 0.002 to 2.8% of the CD4+ T cell pool and did not require *de novo* protein synthesis (59). Studies analyzing the peptide repertoires associated to HLA class II in NK as compared to DC would shed light on these issues. Antigen presentation by NK cells induced a polyfunctional CD4+ T cell activation, qualitatively resembling that detected when using moDC as APCs, characterized by the production of Th1 cytokines and the secretion of their cytotoxic granules, in a fraction of activated cells. Since we only monitored IFNγ and TNFα production, main anti-viral cytokines dominating HCMV-specific CD4+ T cell responses, we cannot exclude the capacity of NK cells for also activating the minority of IL-4, IL-17, or IL-10-producing HCMV-specific CD4+ T cells reported in some individuals (11, 12, 60).

Regarding the consequences of the cognate interaction between autologous cytotoxic CD4+ T lymphocytes and antigen-presenting NK cells, the latter did not degranulate in co-culture experiments ruling out their activation. On the other hand, whether cytotoxic CD4+ T cells may specifically kill antigen-loaded HLA-DR+ NK cells is conceivable yet the possibility that adaptive NK cells may be resistant to the CD4+ T cell cytolytic machinery is not ruled out, as reported for the interaction between memory CD8+ T cells and DC (61).

An additional open question is where antigen presentation by NK cells to specific effector memory CD4+ T cells may take place and to what extent this mechanism could contribute to HCMV infection control. It is tempting to speculate that antigen presentation by NKG2C+ adaptive NK cells may take place at the site of viral reactivation since antigen availability and the chemokine receptor profile expressed by adaptive NK and effector memory CD4+ T cells (CCR7-, CXCR3^low^, CX3CR1+) might preferentially direct them to non-lymphoid/inflamed tissues. Even though performing less efficiently than professional APCs, NK cell-mediated HLA class-II dependent antigen presentation could promote CD4+ T cell responses to HCMV, counteracting its ability to infect professional APC (i.e., dendritic cells or macrophages) (62, 63).

In summary, we have described a novel facet of HCMV-induced NKG2C+ adaptive NK cells residing in their capacity to present antigens to HCMV-specific CD4+ T cells in an HLA-DR-dependent manner, triggering a polyfunctional activation of the cytotoxic effector memory T cell pool.

## Author Contributions

MC-G carried out the experimental work and wrote an initial draft. MA collaborated in the analysis of publically available expression data and experimental work. MM and CV analyzed HLA class II genotypes. AM and ML-B contributed to the design, follow up and interpretation of the results, and wrote the final draft that was revised by all authors.

### Conflict of Interest Statement

The authors declare that the research was conducted in the absence of any commercial or financial relationships that could be construed as a potential conflict of interest.
