# Supplementary material for: Human Cytomegalovirus Antigen Presentation by HLA-DR+ NKG2C+ Adaptive NK Cells Specifically Activates Polyfunctional Effector Memory CD4+ T Lymphocytes
Source: Front Immunol. 2019 Apr 3;10:687. doi: 10.3389/fimmu.2019.00687 (PMC6456717; doi:10.3389/fimmu.2019.00687)
Supplement: Supplementary file 1 [file Image_1.pdf]

A

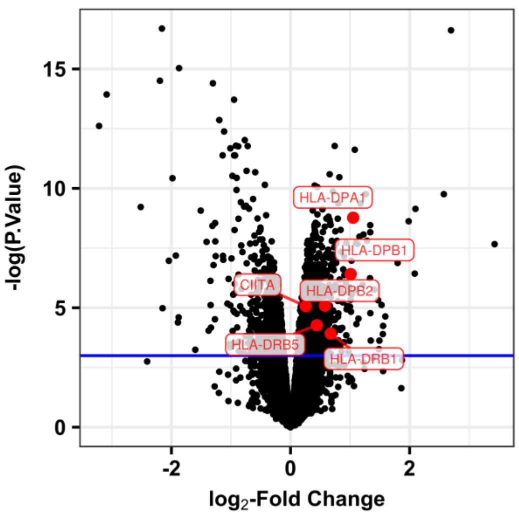

B

Gene symbol	Fold Change
HLA-DRB5	13.7
HLA-DQB1	12.2
HLA-DQA2	11.4
HLA-DQA1	10.6
CIITA	9.8
HLA-DQA1	8.2
HLA-DRB4	7.1
HLA-DRA	6.8
HLA-DRB1	3.8
HLA-DPB1	3.3
HLA-DPB2	3.1
HLA-DPA1	2.7
HLA-DRB3	2.6
HLA-DMA	2.3
CD74	2.3

Supplementary figure 1: Differential expression of genes involved in antigen presentation by MHC class II in adaptive NKG2C+ NK cells. Publicly available gene expression data was obtained from NIH GEO Accession GSE66564 (24) and GSE66124 (25). Already normalized data was log2 transformed, linear model was designed using R limma package and a moderated t-test between the two population groups groups was applied for paired-wise comparison using eBayes function. A volcano plot of the modelled data was generated using the ggplot2 package.

A) Re-analysis of the available microarray data published in Schlums et al. Immunity. 2015. Volcano plot showing fold-change of gene expression in NKG2C+ CD57^{bright} compared to NKG2A+ CD57– CD56^{dim} NK cells. Blue horizontal line indicates a p-value of 0.05 and selected genes are labeled. B) Table extracted from the gene expression microarray analysis data published by Lee et al. Immunity. 2015. comparing sorted NKG2C+ versus NKG2C– NK cells from a donor displaying expansions of adaptive NK cells.

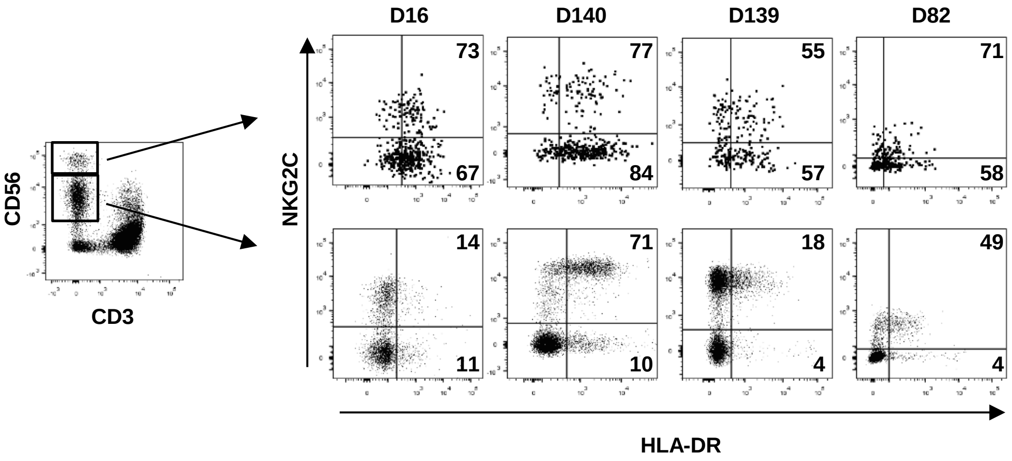

Supplementary figure 2: NKG2C and HLA-DR co-expression in CD56^{bright} and CD56^{dim} NK cells. Representative dot plots of NKG2C and HLA-DR expression in circulating CD56^{bright} and CD56^{dim} NK cells from seropositive individuals with adaptive NKG2C+ NK cell expansions. Inset numbers indicate the percentage of HLA-DR+ cells in NKG2C+ and NKG2C- subpopulations.

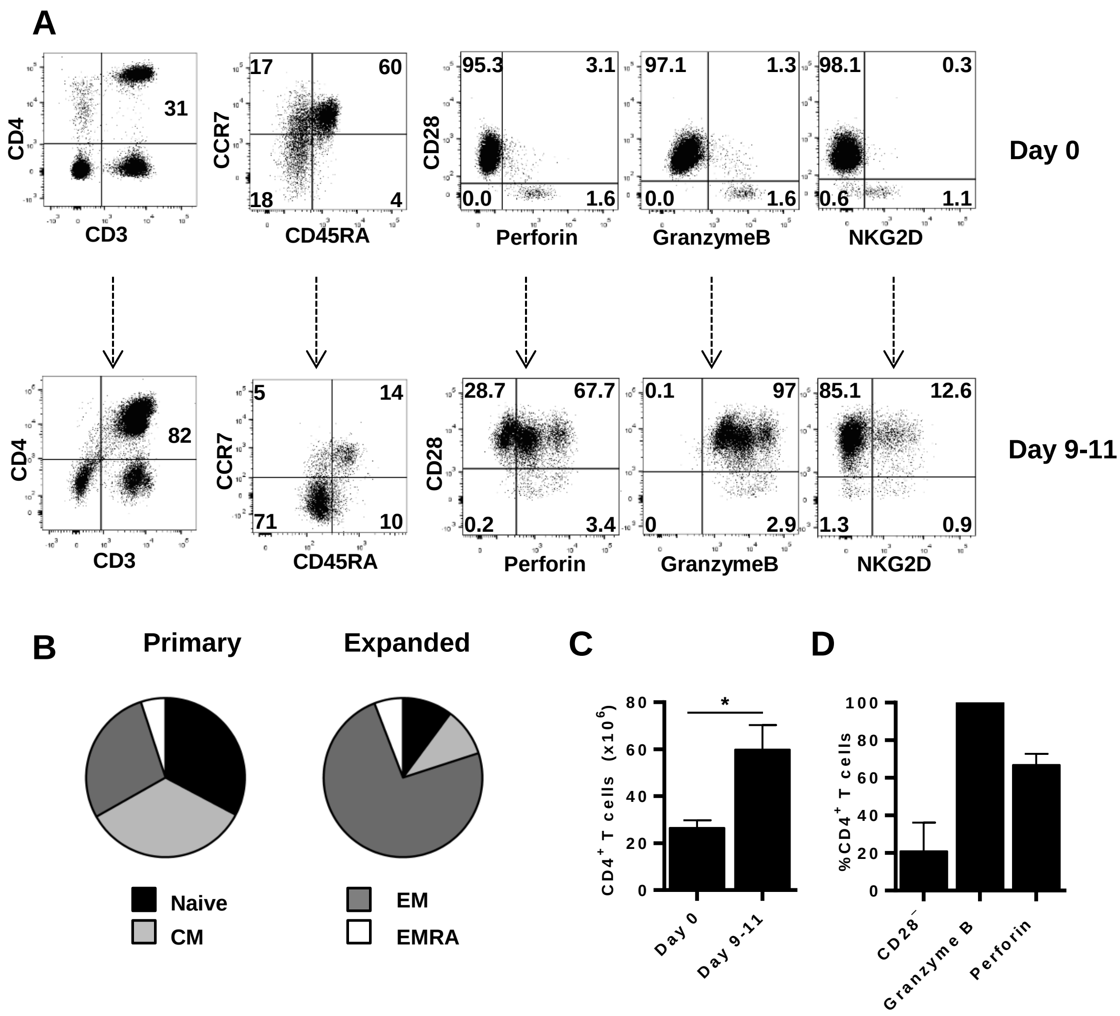

Supplementary Figure 3. Cytotoxic CD4+ T cells expand upon PBMC co-culture with HCMV. CD4+ T cells were expanded by co-culturing PBMC from seropositive donors with HCMV particles (MOI = 0.3) for 9-11 days with 25 U/ml IL-2. **A**) Dot plots showing CD45RA, CCR7 and CD28 expression versus perforin, granzyme B and NKG2D in CD4+ T cells prior and after stimulation with HCMV. Data correspond to a representative expansion. **B)** Pie charts showing the distribution of T cell subpopulations based on CCR7 and CD45RA expression prior and after expansion (mean, n=4). **C)** Average CD4+ T cell numbers prior and after expansion (mean+SEM, n=6). **D**) Frequency of CD28–, perforin+ and granzyme B+ CD4+ T cells after expansion (mean+SEM, n=4).

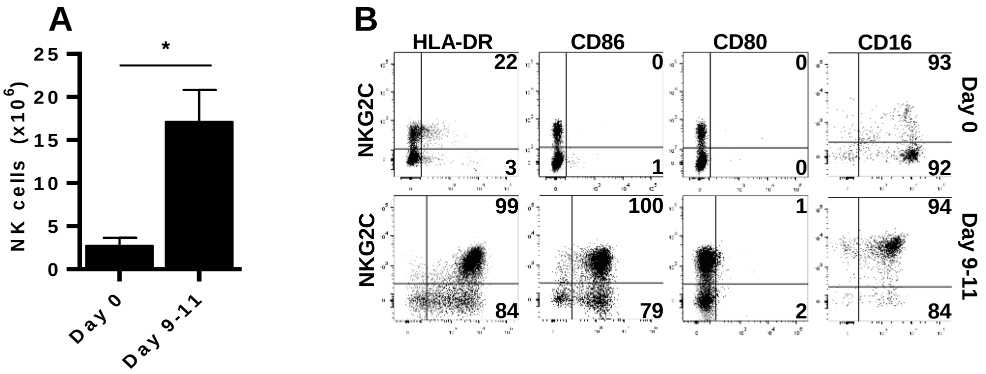

Supplementary Figure 4. NKG2C+ HLA-DR+ NK cell expansion with the HLA-E+ 721.221-AEH cell line. PBMC were cultured with irradiated 721.221-AEH lymphoblastoid cells for 9-11 days. **A**) Average NK cell numbers obtained upon expansion (mean+SEM, n=4). **B**) Dot plots displaying HLA-DR, CD86, CD80, CD16 and NKG2C in NK cells at days 0 and 9-11 (*p < 0.05).

Donor	HLA-DRB1-1	HLA-DRB1-2	HLA-DQB1-1	HLA-DQB1-2	HLA-DPB1-1	HLA-DPB1-2	%TNFα+ IFNγ+ NK	%TNFα+ IFNγ+ DC
**D16**	11:04		03:01		02:01	04:02	0.23	0.6
**D28**	07:01		02:02	03:03	04:01	13:01	0.061	0.45
**D67**	07:01	13:02	02:02	06:04	01:01	02:01	0.43	1.22
**D117**	07:01	11:01	02:02	03:01	04:01		0.32	2.42
**D104**	03:01	07:01	02:01	02:02	04:01	04:02	0.744	
**D82**	03:01	08:02	02:01	04:02	04:01	02:01	0.35	1.48

Supplementary Table I. HLA class II genotype in individuals displaying expansions of HLA-DR+NKG2C+ adaptive NK cells. Last two columns indicate the percentage of CD4+ T cells producing TNFα and IFNγ in antigen-presentation assays using NK cells or DC as APCs.
