# Supplementary material for: Human Cytomegalovirus Antigen Presentation by HLA-DR+ NKG2C+ Adaptive NK Cells Specifically Activates Polyfunctional Effector Memory CD4+ T Lymphocytes
Source: Front Immunol. 2019 Apr 3;10:687. doi: 10.3389/fimmu.2019.00687 (PMC6456717; doi:10.3389/fimmu.2019.00687)
Supplement: Supplementary file 2 [file Data_Sheet_1.PDF]

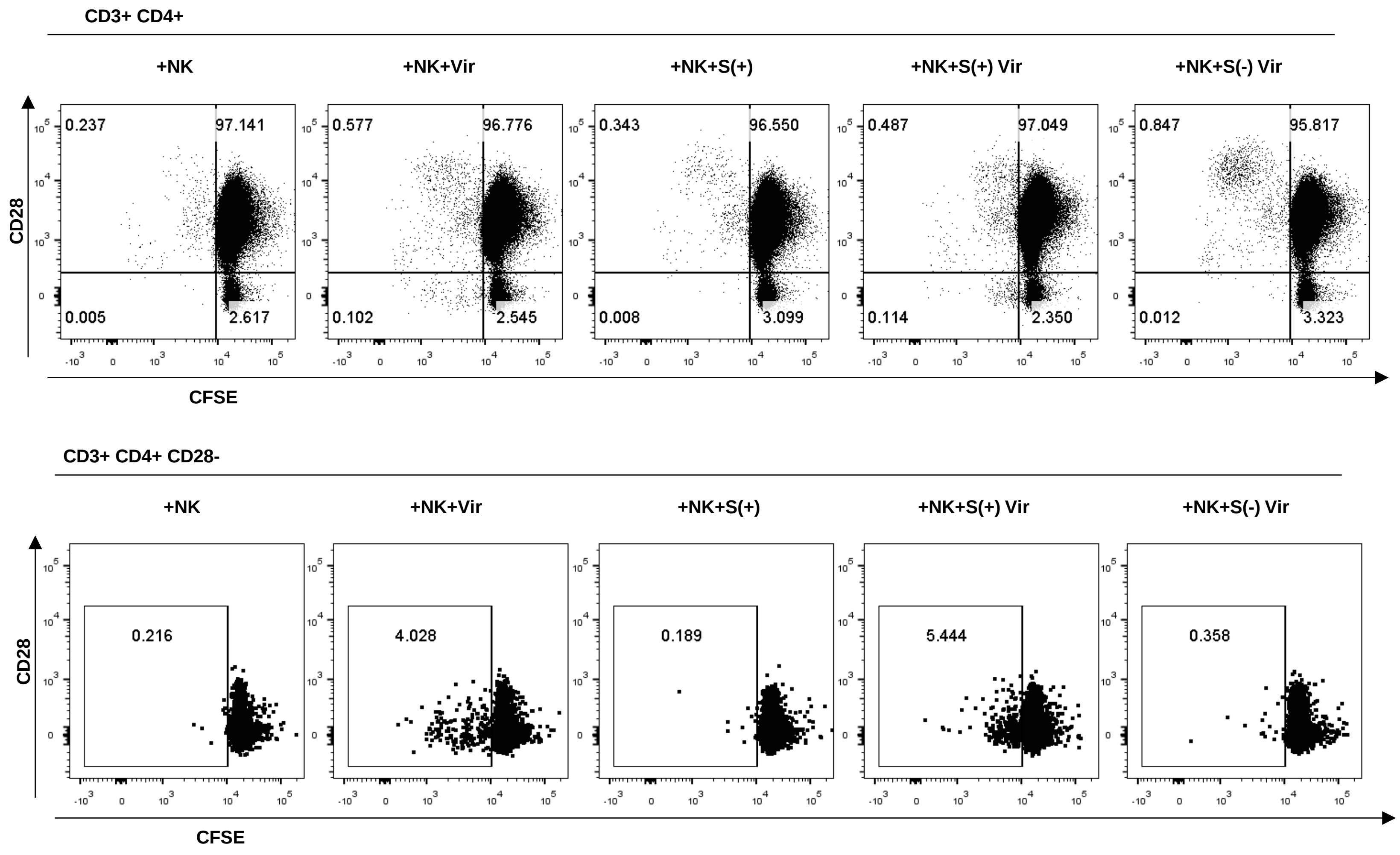

Proliferation of CD4+ CD28- cells upon incubation with antigen loaded NK cells. CFSE-stained CD4+ T cells were activated by NK cells loaded with HCMV in the presence or absence of HCMV+ or HCMV- donor serum and incubated 5 days with 500U/ml of IL2.
